# Strong Nucleating Effect of Si-Containing Tri-Block Oligomers on Poly(Ethylene Terephthalate)

**DOI:** 10.3390/molecules30153077

**Published:** 2025-07-23

**Authors:** Quankai Sun, Yao Wang, Miaorong Zhang, Linjun Huang, Pengwei Zhang, Kang Li, Wei Wang, Jianguo Tang

**Affiliations:** Institute of Hybrid Materials, National Center of International Research for Hybrid Materials Technology, National Base of International Science & Technology Cooperation, College of Materials Science and Engineering, Qingdao University, Qingdao 266071, China; sunquankai11@163.com (Q.S.); zhangmrzhang2018@qdu.edu.cn (M.Z.); newboy66@126.com (L.H.); pengweiz0820@163.com (P.Z.); ascheh@163.com (K.L.); wangwei040901@163.com (W.W.)

**Keywords:** polyesters, silane coupling agent, nucleating agent, crystallization properties

## Abstract

The development of a silane coupling agent with an aminopropyl structure as a nucleating agent for poly(ethylene terephthalate) (PET) is reported in this study. The tri–block oligomers nucleating agent was formed by 1,3-bis(3-aminopropyl)-1,1,3,3-tetramethyldisiloxane/oxalic acid/low molecular weight PET (LPOBD). It was subsequently cross-linked with tetraethyl orthosilicate to form LPOBD-T. Composites of LPOBD/PET and LPOBD-T/PET were prepared by melt blending, and their thermal and crystallization behaviors were analyzed using XRD, DSC, TG, and POM. The results indicated that not only did the triblock polymer nucleating agent LPOBD exhibit a strong nucleation effect, but the crosslinked LPOBD-T also demonstrated superior crystallization performance. Specifically, the crystallinity of the 1 wt% LPOBD-T/PET composite increased by 3.3%, the crystallization temperature rose by 21.1 °C, and the t_1/2_ was reduced by 53 s. Moreover, the crystalline morphology was more uniform. These findings indicate that the tri-block oligomers synthesized from a silane coupling agent serve as effective nucleating agents for PET.

## 1. Introduction

Poly(ethylene terephthalate) (PET) is a widely used thermoplastic polymer renowned for its excellent mechanical properties, chemical stability, and transparency [1,2]. These characteristics make PET a material of choice in textiles [3], packaging [4], and electronics [5]. However, its slow crystallization rate and limited crystallinity challenges for further performance enhancement [6,7]. To address these limitations, researchers have explored various nucleating agents [8,9], including aluminum (Al) [10,11], graphene oxide (GO) [12], silica (SiO_2_) [13], metal-organic frameworks (MOFs) [14] and other materials [15,16,17,18,19]. While these agents aim to accelerate PET’s crystallization rate and improve processability, traditional nucleating agents often suffer from insufficient compatibility with the PET matrix, uneven dispersion, or high loading requirements (>1 wt%) [19,20]. For instance, metallic nucleating agents may agglomerate due to differences in interfacial polarity, while GO [21], despite enhancing crystallinity, may compromise the transparency of the material. Consequently, developing novel nucleating systems that combine high compatibility and low loading remains a critical challenge in this field.

In recent years, silane coupling agents have emerged as a focal point in the research domain of nucleating agents [22,23]. These organosilicon compounds, characterized by the co-presence of silicon atoms and organic functional moieties, assume a pivotal role in augmenting the interfacial compatibility between inorganic fillers and organic polymeric matrices [24,25,26]. Earlier investigations have demonstrated that nano-silica modified via grafting of flexible chain segments serves as an effective nucleating agent for PET [27,28]. The functionalized nano-silica notably enhanced both the crystallization temperature of PET and the spatial homogeneity of dispersed particles. Specifically, the mean particle diameter within the PET matrix decreased from 300 nm to 100 nm, concomitant with a 12.3 °C elevation in crystallization temperature. Notwithstanding, the relatively high loading ratio(2 wt%) necessitates further optimization. Compared with nano-SiO_2_, the silicon-based nucleating agent centered on silane coupling agents exhibits superior nucleating performance. Over the past two years, tetraethyl orthosilicate (TEOS)-derived crosslinked nucleating agents have been developed to address this limitation [29]. However, when incorporated at a loading of 0.75 wt%, these agents induced only marginal changes in the crystallization temperature—specifically, an increase of 7.1 °C. This observation underscores the need for innovative nucleation strategies. It is hypothesized that nucleating agents integrating silane coupling moieties with grafted flexible blocks hold substantial promise, as they are anticipated to markedly enhance PET crystallization performance at a low loadings (≤1 wt%).

A publication ever indicated that 3-aminopropyltriethoxysilane (KH-550) can significantly enhance the compatibility between the inorganic filler CaCu_3_Ti_4_O_12_ and the polyurethane matrix [30]. Similarly, Lee et al. [31] reported that amino-propyl functionalized polyhedral oligomeric silsesquioxanes (A-POSSs) outperformed their non-functionalized counterpart (N-POSS) in elevating the crystallization temperature and shear-induced crystallization rate of PET. The amino-propyl groups in A-POSS established chemical couplings with PET chains. Through the chemical bonding, the A-POSSs achieved a more homogeneous dispersion in the PET matrix [32].

The performance of amino-functionalized A-POSS underscored the potential of amino groups in promoting nucleation. This finding has spurred interest in amide-based nucleating agents [33], which are recognized for enhancing the crystallization temperature and rate of PET by providing heterogeneous nucleation sites [34]. For instance, Luo et al. [35] documented that the presence of amide bonds in polyamide nucleating agents significantly accelerates the heterogeneous nucleation rate of PET. However, despite their ability to induce ordered nucleus formation in PET, aromatic amides typically exhibit poor compatibility with the PET matrix. This incompatibility leads to issues such as migration and precipitation during high-temperature processing or prolonged use, thereby diminishing their nucleation efficiency [36,37]. Given the positive impact of amide groups on nucleation, integrating them into silane coupling agents emerges as a promising strategy [38]. Silane coupling agents can address the dispersion challenges associated with amide groups, while the siloxane network offers enhanced thermal stability, potentially improving the heat resistance of amide bonds.

Herein, this study reports the successful synthesis of a tri-block flexible nucleating agent using a silane coupling agent with solely aminopropyl structures, and investigates its effect on the crystallization properties of PET. On this basis, TEOS was employed for crosslinking modification to validate its impact on crystallization performance. We used 1,3-bis(3-aminopropyl)-1,1,3,3-tetramethyldisiloxane (BATD) to synthesize the nucleating agent. Low-molecular-weight PET (LMPET) was synthesized via the sol-gel method, followed by the preparation of a BATD/acetic acid/LMPET flexible tri-block oligomer nucleating agent (LPOBD). Additionally, TEOS was incorporated into the system to form a non-flexible block nucleating agent (LPOBD-T). Finally, LPOBD and LPOBD-T were melt-blended with pure PET to fabricate composites at different loading ratios, and their properties were systematically evaluated.

## 2. Results and Discussion

### 2.1. Synthesis and Characterization of OBD, LPOBD, and LPOBD-T

**Figure 1:**
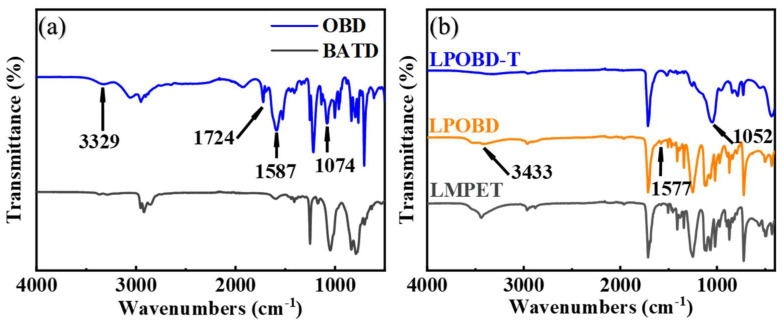
FTIR spectra of (**a**) BATD and OBD and (**b**) LMPET, LPOBD and LPOBD-T.

OBD, LPOBD and LPOBD-T were analyzed by FTIR. The absorption peak observed at 1587 cm^−1^ in Figure 1a corresponds to the in-plane bending vibrations of the N–H group in the amide bond. The absorption peak at 1724 cm^−1^ represents the stretching vibration of C=O. The absorption peak at 3329 cm^−1^ is widened and strengthened, which was caused by the combined effects of the stretching vibration peaks of –OH and –N–H. In addition, the absorption peak around 1074 cm^−1^ became wider and more complex, which was caused by the combined effect of C–O and Si–O stretching vibration peaks [39]. The results indicate that the –COOH group in oxalic acid reacted with the –NH_2_ group in BATD. 

As can be observed in Figure 1a, the –OH stretching vibration peak at 3433 cm^−1^ on the LPOBD infrared absorption curve is less sharp than that on the LMPET absorption curve, indicating that OBD had reacted with PET and consumed part of the –OH. At the same time, the N–H in-plane bending absorption peak at 1577 cm^−1^ can also prove that OBD had bound to PET [40,41]. With the addition of TEOS, the –OH absorption peak at 3433 cm^−1^ in the LPOBD-T spectrum was further weakened, and the Si–O stretching vibration peak at 1052 cm^−1^ became wider, proving that LPOBD had reacted with TEOS.

**Figure 2:**
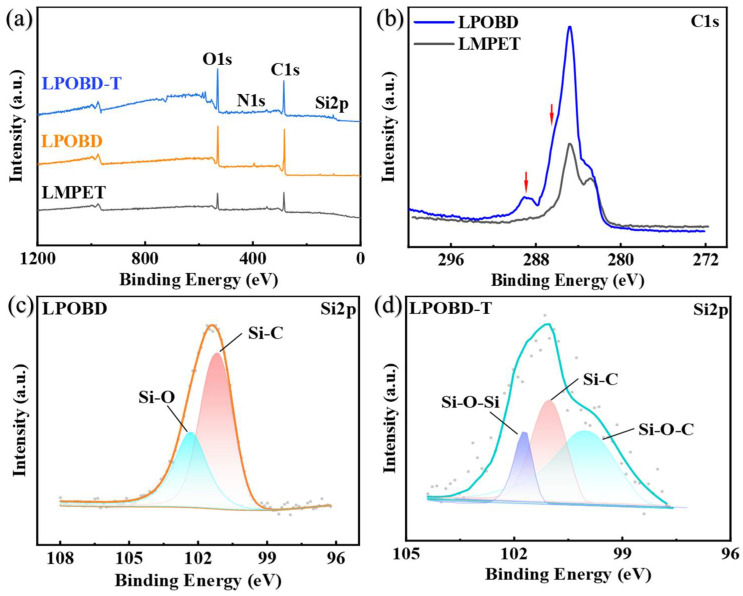
(**a**) XPS spectra of LMPET, LPOBD and LPOBD-T; (**b**) C1s spectra of LPOBD; (**c**) Si2p spectra of LPOBD; (**d**) Si2p spectra of LPOBD-T.

XPS analysis can clarify the valence bonds of the samples. The total spectrum is shown in Figure 2a, where the LMPET has only two peaks: C and O. The LPOBD and LPOBD-T show distinct N and Si peaks on the spectra. Additionally, compared to LMPET, the C1s spectrum of LPOBD (Figure 2b) shows distinct peaks at 286.11 eV and 288.73 eV, with LPOBD exhibiting higher absorption intensity. These two peaks correspond to the absorption peaks of C–N and C=O, respectively, indicating that the –COOH in OBD reacted with the –OH in LMPET, which led to an increase in the C=O content. The increase in both C–N and C=O can also be attributed to the amide bond in OBD [42]. Therefore, we conclude that OBD has reacted with LMPET to form LPOBD.

The successful synthesis of LPOBD-T can be reflected by the characteristic peaks of Si2p of LPOBD and LPOBD-T. Compared to Figure 2c, the Si–O–C peak appeared and the intensity of the Si–C peak became weakened as can be seen in Figure 2d, indicating a reduction in Si–C bonds on the material surface [31]. It can be inferred that the molecular clusters produced by the TEOS are more abundant on the material surface. Thus, some characteristic peaks of Si–C are obscured. 

Additionally, high-resolution scanning electron microscopy (HR-SEM) images of LMPET, LPOBD, and LPOBD-T are presented in Figure S4a. Comparative analysis shows that LMPET exhibits a fluffy structure, while the structure of LPOBD became denser after introducing OBD. These phenomena can be attributed to the role of amide bonds: Nitrogen atoms in amide bonds form hydrogen bonds with C=O groups in LMPET, enhancing inter-molecular attraction and promoting tighter packing of molecular chains. The surface of LPOBD-T displays abundant clusters, resulting from the dense crosslinked network formed by TEOS. This observation aligns with the XPS analysis results. Figure S4b,c illustrate the elemental mapping of LPOBD and LPOBD-T, showing uniform dispersion of Si and N elements. LPOBD-T exhibits a more intensive Si distribution, further confirming the successful synthesis of LPOBD and LPOBD-T.

### 2.2. Hybrid PET Materials Doped by LPOBD and LPOBD-T

**Figure 3:**
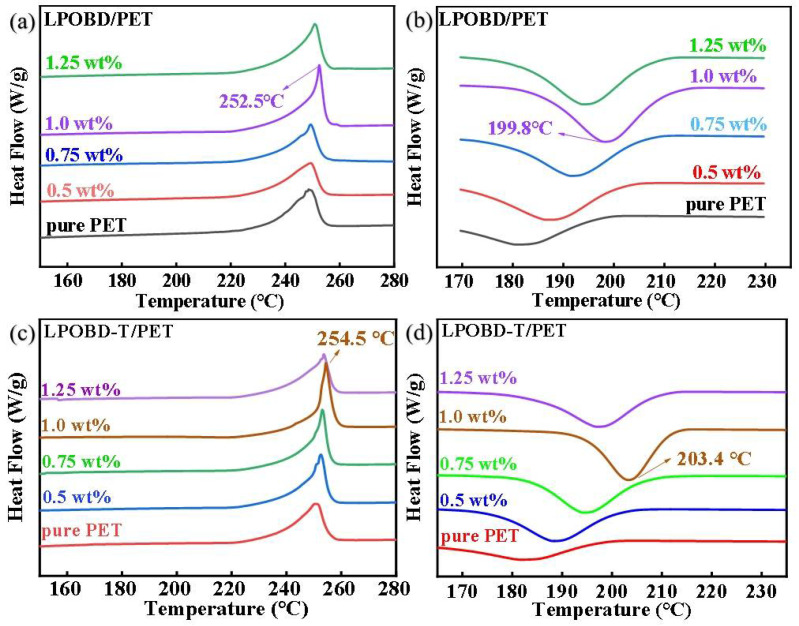
DSC curves of PET composites at a rate of 10 °C/min: (**a**,**c**) heating curves; (**b**,**d**) cooling curves.

The heating curves and cooling curves of the three materials obtained by DSC tests are shown in Figure 3. The DSC data for different material compositions and cooling rates are presented in Tables S3 and S4. The formula for calculating crystallinity (Xc) is consistent with that of Ge et al. [43].

As revealed by Figure 3a,b and Table 1, the melting temperature (Tm) and crystallization temperature (Tc) of LPOBD/PET composites exhibit a trend of first increasing and then decreasing as the nucleating agent loading increases. When the loading was below 1 wt%, Tm and Tc of the PET composites gradually increased with increasing LPOBD loading. These temperatures reached their maximum values at a loading of 1 wt%. The Tm of the PET increased from 248.7 °C to 252.5 °C, and the Tc rose from 182.3 °C to 199.8 °C. Additionally, the Xc increased by 3%, and the half-crystallization time (t_1/2_) shortened by 34 s. The values of melting enthalpy (ΔHm) and crystallization enthalpy (ΔHc) also show an upward trend. However, when the loading exceeded 1 wt%, both the Tm and Tc decreased instead.

**Figure 4:**
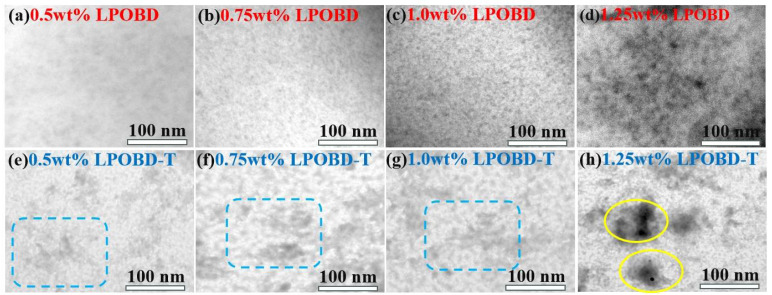
TEM images of: (**a**–**f**) LPOBD/PET composites and (**e**–**h**) LPOBD-T/PET composites.

This phenomenon is closely associated with the dispersion status of nucleating agents in the PET matrix. The distribution of dark particles gradually became denser with the increase in LPOBD content (Figure 4a–c), and the LPOBD exhibited good dispersion at a loading of 1 wt%, as shown in Figure 4c. When the loading content increased to 1.25 wt%, obvious massive agglomeration occurred in the PET matrix, as shown in Figure 4d. This indicates that an increase in LPOBD concentration provides more nucleation sites, promoting heterogeneous nucleation of PET and thereby enhancing crystallinity. The higher the crystallinity, the more ordered molecular chain regions exist in the polymer, leading to stronger intermolecular forces. As a result, PET requires higher energy to transform from the ordered state to the disordered melt state, causing an increase in the Tm. However, when the LPOBD loading exceeds 1 wt%, excessive LPOBD agglomerates in the PET matrix, reducing effective nucleation sites and impairing crystallization.

The dispersion behavior of LPOBD in the PET matrix can be attributed to inter-molecular hydrogen bonding. The amide bonds in LPOBD molecules enable hydrogen bonding with both the PET matrix and other LPOBD molecules. At a low loading (<1 wt%), the large inter-molecular distances promote interactions between LPOBD and PET, resulting in uniform dispersion. At 1 wt%, these interactions saturate. Above this concentration, spatial limitations decrease inter-molecular distances among LPOBD molecules, leading to self-association and agglomeration.

Furthermore, the characteristics of LPOBD-T/PET composites overall mirror the trend observed in LPOBD/PET composites. However, LPOBD-T/PET composites exhibit superior performance. Specifically, as shown in Figure 3c,d and Table 1, the Tm of PET increased from 248.7 °C to 254.5 °C, and the Tc rose from 182.3 °C to 203.4 °C. The Xc increased by 3.3%, and the t_1/2_ shortened by 53 s. Similarly to LPOBD, the dispersion state of LPOBD-T (Figure 4e–h) reached saturation at a loading of 1 wt% and showed massive agglomeration with particle sizes >100 nm at a loading of 1.25 wt%. Notably, LPOBD-T displays a distinct crosslinked morphology within the PET matrix. These results demonstrate that TEOS-mediated crosslinking enhanced the crystallization capability of the flexible tri-block nucleating agent LPOBD without compromising the uniform dispersion of the nucleating agent.

**Figure 5:**
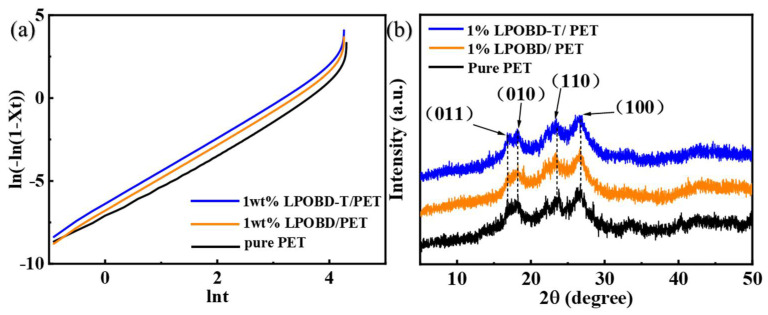
(**a**) The logarithm Avrami diagram ln[−ln(1 − X_t_)] versus ln(t) and (**b**) X-ray diffraction patterns of 1 wt% PET composites.

The crystallization kinetic behavior of PET composites has a significant impact on their applications. The crystallization process of most crystalline polymers can be described using the Jeziorny method and Avrami equation [44,45], and the formula results are shown in Figure 5a and Table 1.

The Avrami plots of the composites all exhibit linear segments initially in the early stage of crystallization, and the Avrami index (n) ranges from 2 to 3. These results suggest that the addition of LPOBD and LPOBD-T does not alter the crystal growth mechanism of PET [46]. 

The XRD patterns of pure PET, 1 wt% LPOBD/PET, and 1 wt% LPOBD-T/PET after annealing under the same conditions are shown in Figure 5b. All quantitative analysis results are presented in Table S5. It can be observed that the three types of PET exhibit very similar interplanar spacing (d*_hkl_*) values for all relevant crystallographic planes—(011), (010), (110), and (100)—due to their identical triclinic crystal system [27,47]. This indicates that the unit cell parameters of the triclinic crystal lattice of PET remain unchanged during its heterogeneous nucleation process. Furthermore, the crystallite size (L*_hkl_*) values of the PET matrix show almost no variation on each crystallographic plane after the incorporation of small amounts of LPOBD or LPOBD-T. Overall, in terms of crystal form, unit cell geometry, and crystallite size, the introduction of LPOBD or LPOBD-T causes minimal disruption to the crystal structure of PET. This suggests that heterogeneous nucleation essentially does not affect the subsequent crystal growth mechanism of the PET matrix. These results are consistent with the conclusions derived from the Avrami equation.

**Figure 6:**
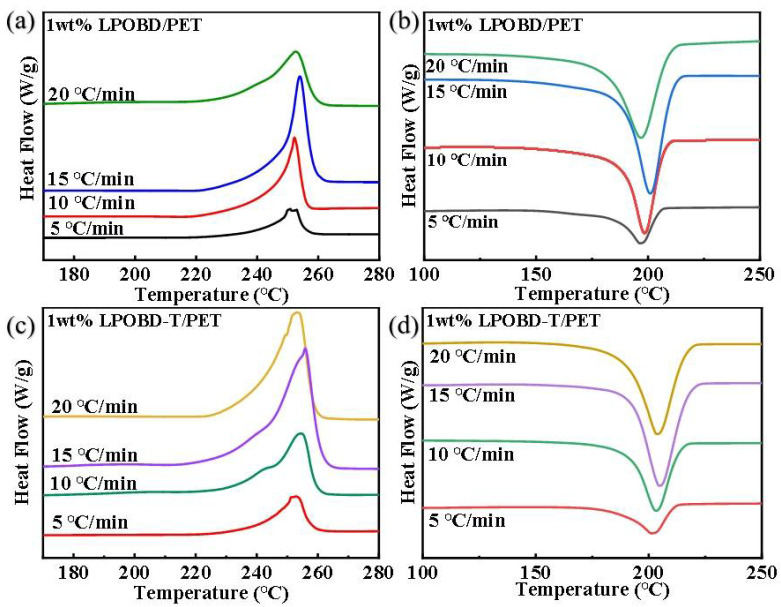
(**a**) Heating curves and (**b**) cooling curves of 1 wt% LPOBD/PET composites at different cooling rates; (**c**) heating curves and (**d**) cooling curves of 1 wt% LPOBD-T/PET composites at different cooling rates.

**Figure 7:**
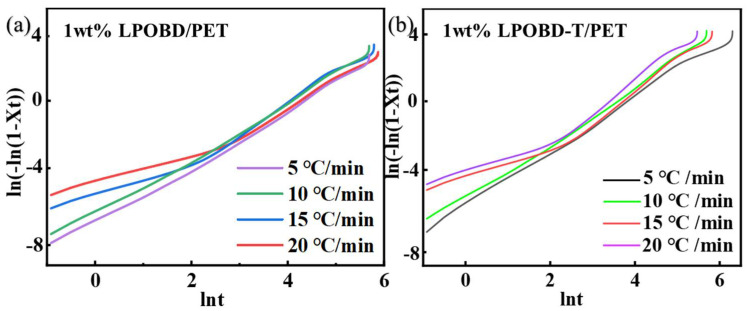
The logarithm Avrami diagram ln[−ln(1 − Xt)] versus ln(t) curves of (**a**) 1 wt% LPOBD/PET composites and (**b**) 1 wt% LPOBD-T/PET composites at different cooling rates.

For 1 wt% PET composites, DSC curves at different rates were used to further investigate their crystallization behavior. As shown in Figure 6 and Tables S6 and S7, the PET composites exhibited the highest values of Tm, Tc, ΔHm, and ΔHc when crystallized at a rate of 15 °C/min. However, as depicted in Figure 7, the n value deviated from linear behavior at the initial stage under a 15 °C/min rate. This result indicates that the crystallization process becomes more complex at 15 °C/min, which is inconsistent with our research objectives. In this case, inter-molecular hydrogen bonding significantly restricts the movement of molecular segments. Thus, we conclude that the crystallization behavior at a rate of 10 °C/min is more worthy of study.

To further study the effect of nucleating agents on the crystallization behavior of PET, polarizing microscope observations were performed on pure PET, 1 wt% LPOBD /PET and 1 wt% LPOBD-T/PET. The crystallization images of the samples cooled from 290 °C at a rate of 10 °C /min are shown in Figure S5. The dark regions in the images represent amorphous areas, while the bright parts correspond to crystalline regions. The real-time temperature and scale of crystallization are placed in the upper left corner and lower right corner of the picture respectively. The crystalline morphology primarily exhibits an interlocking texture of spherulites, as these spherulites are distributed within the amorphous matrix [48,49].

**Figure 8:**
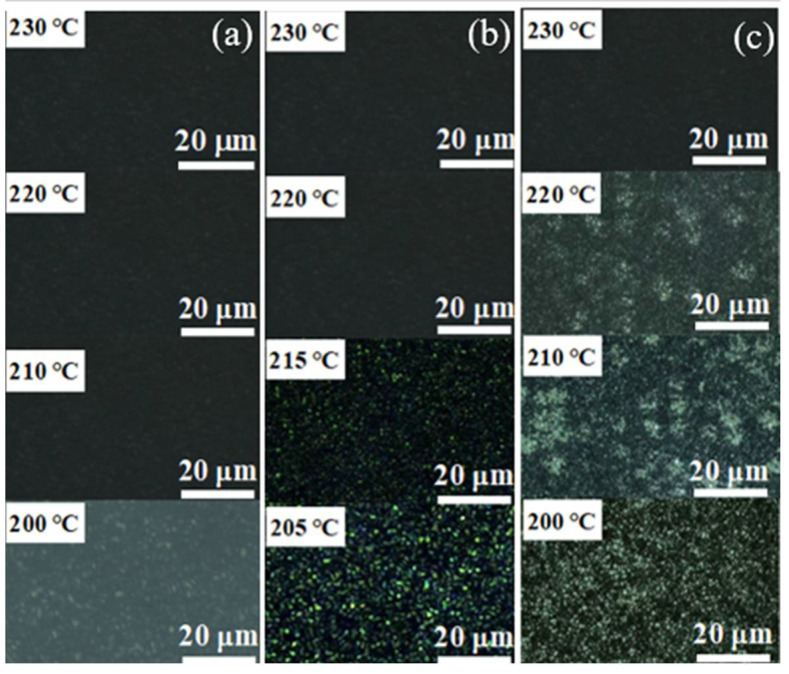
POM images of (**a**) pure PET, (**b**) 1 wt% LPOBD/PET, and (**c**) 1 wt% LPOBD-T/PET.

Through comparison, we observed that the crystalline regions of pure PET appear relatively dim. The crystalline regions of LPOBD and LPOBD-T modified materials are notably brighter, with slightly larger grain sizes. This indicates that the addition of LPOBD and LPOBD-T promotes PET crystallization, resulting in more well-defined crystalline regions [50]. These findings are consistent with the XRD and DSC analyses. Additionally, the onset crystallization temperature of pure PET is 200 °C, whereas those of LPOBD/PET and LPOBD-T/PET composites are 215 °C and 220 °C, respectively (Figure 8). This demonstrates that the incorporation of nucleating agents significantly accelerates the crystallization rate of PET.

Additionally, the LPOBD-T/PET composite exhibits higher crystallinity and brightness in its texture compared to the LPOBD/PET composite, and its onset crystallization temperature is 5 °C higher. This suggests that the crosslinked LPOBD exhibits superior nucleating performance. The TEOS crosslinking positively enhances the effectiveness of the nucleating agent. With the improvement in crystallization performance, the thermal stability of PET composites has also been correspondingly enhanced (Figure S6).In conclusion, the analysis leads to the following key findings: Both LPOBD and LPOBD-T are effective nucleating agents for PET, with LPOBD-T demonstrating superior performance; TEOS crosslinking modification is beneficial for enhancing the nucleating performance of LPOBD. 

## 3. Experimental

### 3.1. Materials

We obtained 1,3-bis(3-aminopropyl)-1,1,3,3-tetramethyldisiloxane (98%) from Leyan.com, dimethyl-p-phthalate (DMT, CP), and ethanol absolute (AR, 99.7%) were purchased from Sinopharm Chemical Reagent Co., Ltd. (Shanghai, China). Tetraethyl orthosilicate (TEOS, 98%), ethylene glycol (EG, 99%), triphe-nylphosphine (TPP, 98%), zinc acetate (Zn(Ac)_2_, 99.99%) and antimony(III) oxide (Sb_2_O_3_, AR, 99.5%) were purchased from Shanghai Macklin Biochemical Technology Co., Ltd. (Shanghai, China). Phenol (AR, 99%), tetrachloroethane (AR, 99%) and oxalic acid dihydrate (99.5%) were purchased from Sinopharm Chemical Reagent Co., Ltd. (Shanghai, China). The commercial PET polyester slices (processing grade: fiber grade, non-extinction, intrinsic viscosity 0.8 dL/g) were purchased from Sinopec Yizheng Chemical Fiber Co., Ltd. (Yizheng, China).

### 3.2. Synthesis of OBD Nanocomposites

A 100 mL of three-necked round-bottomed flask, maintained under a nitrogen atmosphere and equipped with an external condenser, was utilized for the synthesis. The flask was charged with 16.4 g (0.13 mol) of oxalic acid dihydrate and 40 mL of deionized water and stirred at 90 °C. Subsequently, 10.8 mL (0.04 mol) of BATD was introduced dropwise into the mixture within 10 min. After cooling, the resulting mixture was washed two times by centrifugation with deionized water. Finally, the product OBD was dried at 80 °C in a vacuum oven for 48 h. The yield of OBD was 91%.

### 3.3. Synthesis of LPOBD Nanocomposites

LMPET was synthesized using the method reported previously [51]. LMPET and OBD were dried at 80 °C in a vacuum oven for 48 h.

An amount of 28.1 g (0.03 mol) of LMPET was dissolved in a phenol/tetrachloroethylene (mass ratio1:1) solution and stirred at 130 °C for 15 min. Then, 4.2 g (0.01 mol) of OBD was added to the solution and continuously stirred for 1 h. After cooling, the product LPOBD was isolated by sequential rinsing with phenol/tetrachloroethylene (mass ratio 1:1) and absolute ethanol, followed by vacuum drying at 80 °C for 48 h. The yield of LPOBD was 87%. The structure of LPOBD is shown in Figure S1.

### 3.4. Synthesis of LPOBD-T Nanocomposites

38.6 g (0.02 mol) of LPOBD was dispersed in a mixed solution of phenol/ tetrachloroethylene (mass ratio 1:1) and stirred at 130 °C for 15 min. Subsequently, 5 mL of EG and 8.9 mL (0.04 mol) of TEOS [52] were added dropwise to the mixed solution within 10 min and reacted for 1 h. After cooling, the product LPOBD-T was subjected to rinsing with absolute ethanol and subsequently dried in a vacuum oven at 80 °C for 48 h. The yield of LPOBD was 80%. The molecular weight is shown in Table S1. The structure of LPOBD-T is shown in Figure S2.

### 3.5. Synthesis of Doped PET Composites with Both LPOBD and LPOBD-T

LPOBD, LPOBD-T, and pure PET polyester slices were dried in a vacuum oven at 120 °C for 48 h. They were sequentially introduced into a twin-screw microcompound extruder and an injection molding machine. The temperature of the feeding zone was set at 280 °C, while the mold temperature was maintained at 90 °C. Additionally, the injection molding machine temperature was adjusted within the range of 275/285/295 °C. 

**Figure 9:**
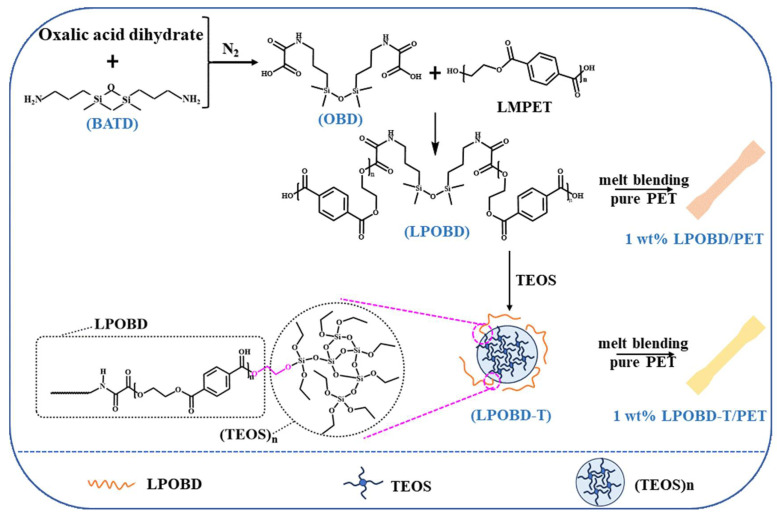
Synthesis routes of hybrid PET materials.

The dimensions of each model were measured with a total length of 75 mm, a narrow parallel side length of 25 mm, a width of 4 mm, and a thickness of 2 mm (Figure S3). In this work, a series of PET hybrid materials with LPOBD and LPOBD-T loadings of 0.5/0.75/1.0/1.25 wt% were successfully prepared, and the effects of different nanostructures on the performance of PET hybrid materials were compared. The synthesis routes are shown in Figure 9 and the abbreviations are shown in Table S9.

### 3.6. Characterization

Fourier Transform Infrared (FT-IR, IS-50, Thermos-fisher, Waltham, MA, USA) and X-ray Photoelectron Spectroscopy (XPS, K-Alpha, Thermos-fisher, USA) were used to characterize the surface functional groups and chemical composition of the samples in the range of 4000–400 cm^−1^. Scanning Electron Microscopy (SEM, JSM-7500 F, JEOL, Tokyo, Japan) and Transmission Electron Microscopy (TEM, JSM-2100 F, JEOL, Tokyo, Japan) were used to observe the surface morphology of LMPET, LPOBD, and LPOBD-T. Nuclear Magnetic Resonance (NMR, AVANCE III HD 500 MHz, Bruker, Germany) was used to characterize the chemical environment of different elements. Gel Permeation Chromatography (GPC, 1260 Infinity II, Agilent, USA) was used to characterize the molecular weights of PET materials.

The crystalline structure of the polymers was tested by X-ray Diffraction (XRD, D8 advance, Bruker AXS, Karlsruhe, Germany). Differential Scanning Calorimetry (DSC, DSC 250, Waters, Milford, MA, USA) was used to study the melting and crystallization of the samples in an N_2_ atmosphere. The temperature was increased from 30 °C to 300 °C at a rate of 5/10/15/20 °C/min, held for 10 minutes, and then cooled to room temperature at the same rate. The heating process was in two cycles. Polarized Optical Microscopy (POM, Axioscope 5, Carl Zeiss, Germany) was used to observe the crystallization behavior of the samples. Finally, Thermogravimetric Analysis (TG, TG 209, Netzsch, Germany) was used for thermal (30–600 °C, 10 °C/min) tests. Other parameters are shown in Table S2.

## 4. Conclusions

This study successfully synthesized a silicon-containing triblock oligomer as a nucleating agent for PET, demonstrating its strong crystallization capability. Compared with previous work, both LPOBD and LPOBD-T significantly promoted crystallization at a loading of 1 wt% (Table S10). Moreover, crosslinked LPOBD-T significantly enhanced the performance of the nucleating agent compared with LPOBD. Specifically, the 1 wt% LPOBD/PET composite exhibited a 3% increase in X_c_, a 17.5 °C rise in T_c_, and a 34 s reduction in t_1/2_. However, the 1 wt% LPOBD-T/PET composite showed a 3.3% increase in X_c_, a 21.1 °C increase in Tc, and a 53s reduction in t_1/2_. Additionally, both LPOBD and LPOBD-T significantly improved the crystalline morphology of PET. These findings indicate that LPOBD and LPOBD-T serve as effective nucleating agents for PET, significantly enhancing its crystallization capability.

## Figures and Tables

**Table 1:** DSC data of 1 wt% PET hybrid materials.

Sample	T_c_ (°C)	T_m_ (°C)	ΔH_m_ (J/g)	ΔH_c_ (J/g)	X_c_ (%)	t_1/2_ (s)	n	K_c_
Pure PET	182.3	248.7	32.1	31.1	22.2	128	2.35	0.26
1% LPOBD	199.8	252.5	36.3	34.9	25.2	94	2.76	0.38
1% LPOBD-T	203.4	254.5	37.8	35.3	25.5	75	2.81	0.43

Notes: T_m_ is the melting temperature; T_c_ is the crystallization temperature; ΔH_m_ is the melting enthalpy; ΔH_c_ is the crystallization enthalpy; X_c_ is the crystallinity; t_1/2_ is the half-crystallization time; n is the Avrami index; Kc is the crystallization rate constant.

## Data Availability

The original contributions presented in the study are included in the article; further inquiries can be directed to the corresponding author.

## Supplementary Materials

The following supporting information can be downloaded at https://www.mdpi.com/article/10.3390/molecules30153077/s1. Figure S1: 1H NMR spectrum of LPOBD; Figure S2: 1H NMR spectrum of LPOBD-T; Figure S3: The Shape of PET hybrid materials; Figure S4: (a) SEM images of LMPET, LPOBD and LPOBD-T, and the Elemental Mapping images of (b) LPOBD and (c) LPOBD-T; Figure S5: POM images of (a) pure PET, (b) 1 wt% LPOBD/PET, and (c) 1 wt% LPOBD-T/PET; Figure S6: Thermal analysis of hybrid PET materials; Table S1: The molecular weight of LMPET, LPOBD, and LPOBD-T; Table S2: The parameters for materials; Table S3: Thermal properties and crystallization kinetics analysis data of LPOBD/PET composites; Table S4: Thermal properties and crystallization kinetics analysis data of LPOBD-T/PET composites; Table S5: The scattering crystallographic plane (hkl), 2θ, dhkl, Lhkl, and Xc values of PET materials; Table S6: Thermal properties and crystallization kinetics analysis data of 1 wt% LPOBD/PET composites at different cooling rates; Table S7: Thermal properties and crystallization kinetics analysis data of 1 wt% LPOBD-T/PET composites; Table S8: Thermal properties of hybrid PET materials; Table S9: Abbreviations and their corresponding full names; Table S10: A comparison between the results of this paper and the published results.
