# Supplementary material for: Strong Nucleating Effect of Si-Containing Tri-Block Oligomers on Poly(Ethylene Terephthalate)
Source: Molecules. 2025 Jul 23;30(15):3077. doi: 10.3390/molecules30153077 (PMC12348685; doi:10.3390/molecules30153077)
Supplement: Supplementary file 1 [file molecules-30-03077-s001.zip › molecules-3753804-supplementary.pdf]

Strong Nucleating Effect of Si−containing Tri−block Oligomers on Poly(ethylene terephthalate）

Quankai Sun[a], Yao Wang[a, **], Miaorong Zhang[a], Linjun Huang[a], Pengwei Zhang[a], Kang Li[a], Wei Wang[a], Jianguo Tang[a, *]

[a] Institute of Hybrid Materials, National Center of International Research for Hybrid Materials Technology, National Base of International Science & Technology Cooperation, College of Materials Science and Engineering, Qingdao University, Qingdao 266071, P. R. China

* Corresponding author.

** Corresponding author.

E−mail addresses: tang@qdu.edu.cn (J. Tang), wangyaoqdu@126.com (Y. Wang).

Figure S1. ^{1}H NMR spectrum of LPOBD.

Figure S2. ^{1}H NMR spectrum of LPOBD−T.

Figure S3. The Shape of PET hybrid materials.

Figure S4. (**a**) SEM images of LMPET, LPOBD and LPOBD−T, and the Elemental Mapping images of (**b**) LPOBD and (c) LPOBD−T.

Figure S5. POM images of (**a**) pure PET, (**b**) 1 wt% LPOBD/PET, and (**c**) 1 wt% LPOBD−T/PET.

Figure S6. Thermal analysis of hybrid PET materials.

Table S1. The molecular weight of LMPET, LPOBD, and LPOBD−T.

Table S2. The parameters for materials.

Table S3. Thermal properties and crystallization kinetics analysis data of LPOBD/PET composites.

Table S4.Thermal properties and crystallization kinetics analysis data of LPOBD−T/PET composites.

Table S5. The scattering crystallographic plane (hkl), 2θ, dhkl, Lhkl, and xc values of PET materials.

Table S6. Thermal properties and crystallization kinetics analysis data of 1wt% LPOBD/PET composites at different cooling rates.

Table S7. Thermal properties and crystallization kinetics analysis data of 1wt% LPOBD−T/PET composites.

Table S8. Thermal properties of hybrid PET materials.

Table S9. Abbreviations and their corresponding full names.

Table S10.A comparison between the results of this paper and the published results.

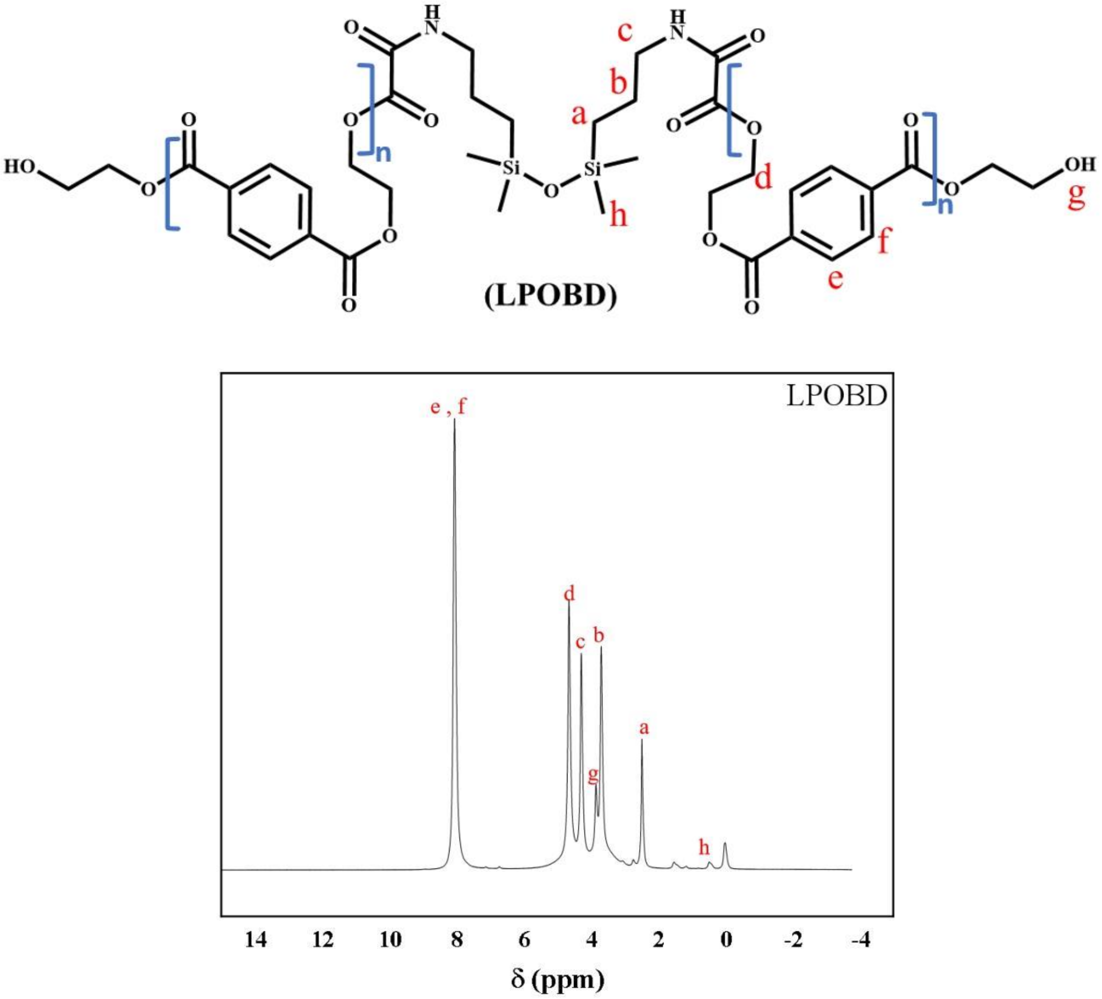

Figure S1. ^{1}H NMR spectrum of LPOBD.

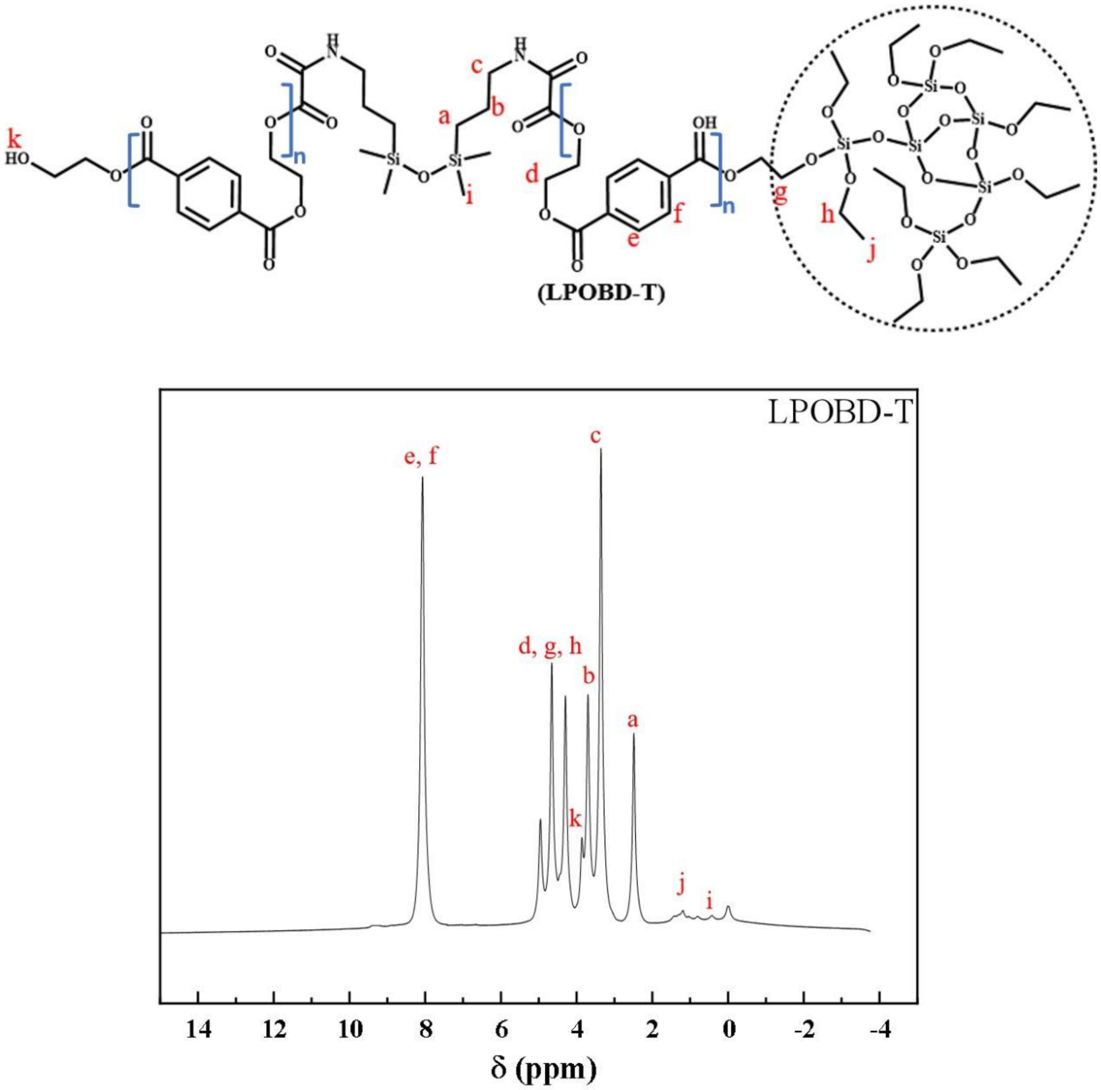

Figure S2. ^{1}H NMR spectrum of LPOBD−T.

Table S1.

The molecular weights of LMPET, LPOBD, and LPOBD−T.

Sample	Mn (g/mol)	Mw (g/mol)	Mw/Mn
LMPET	936	1357	1.45
LPOBD	1930	2576	1.33
LPOBD−T	4283	6167	1.44

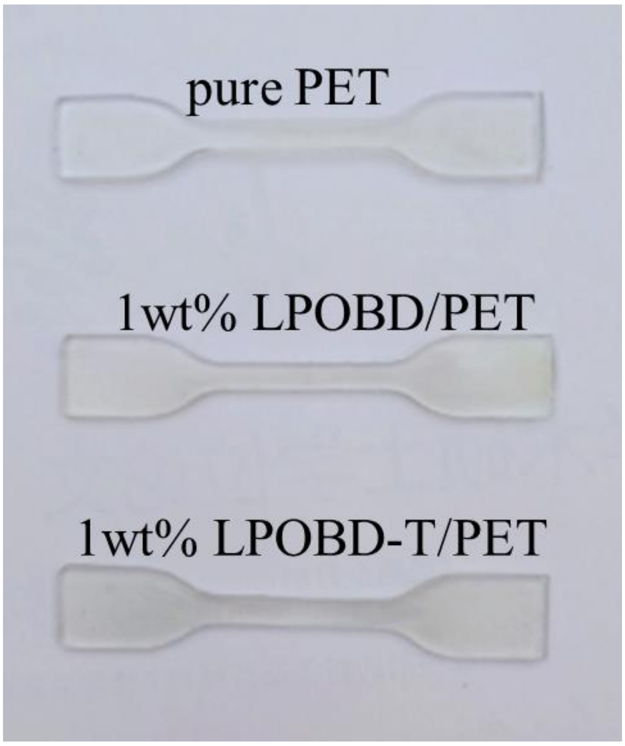

Figure S3. The Shape of PET hybrid materials.

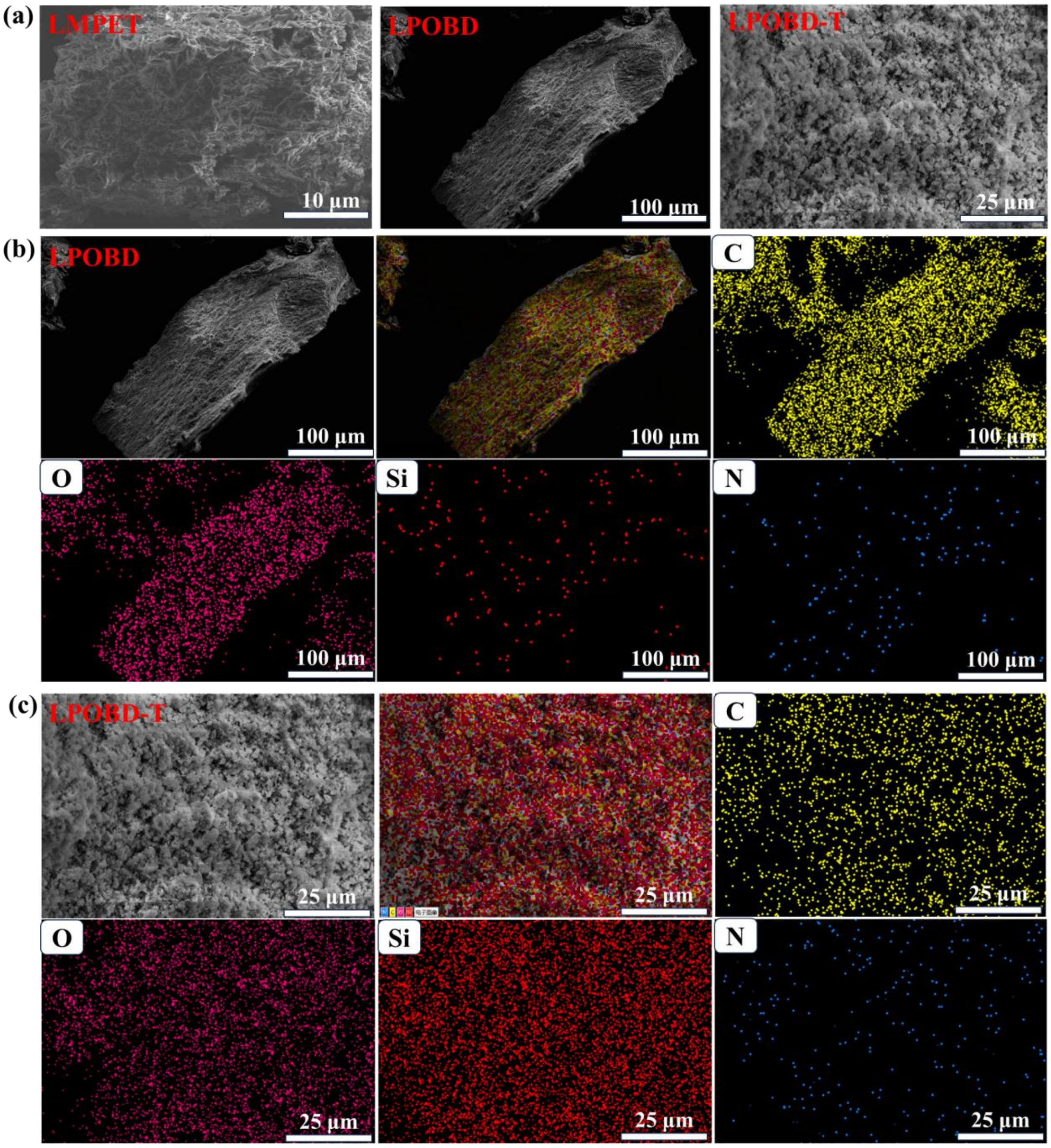

Figure S4. (**a**) SEM images of LMPET, LPOBD and LPOBD−T, and the Elemental Mapping images of (**b**) LPOBD and (c) LPOBD−T.

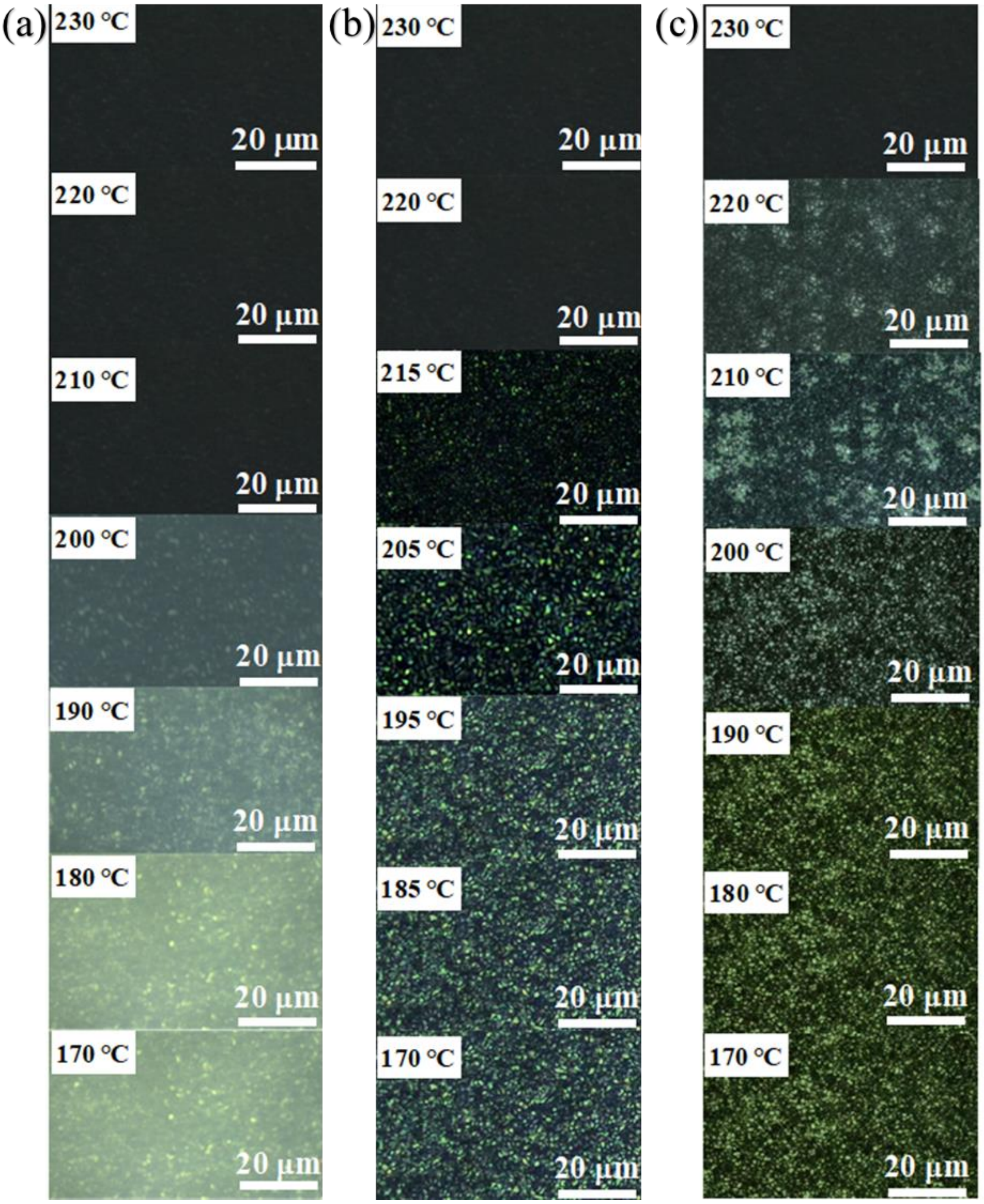

Figure S5. POM images of (**a**) pure PET, (**b**) 1 wt% LPOBD/PET, and (**c**) 1 wt% LPOBD−T/PET.

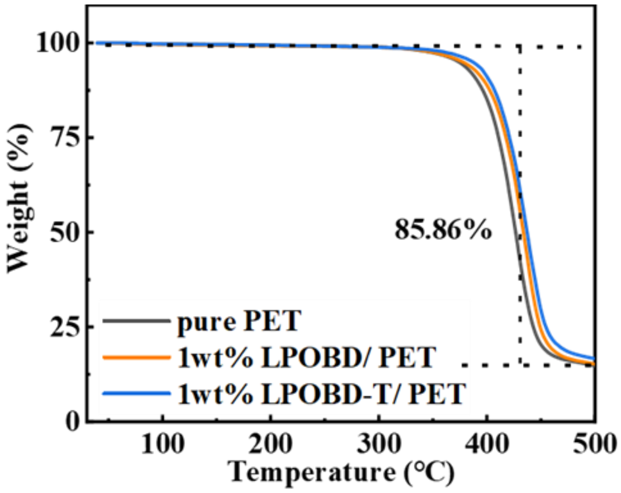

Figure S6. Thermal analysis of hybrid PET materials.

Table S2.

The parameters for materials.

Characterization	Parameters
XRD	5°to 60° with scanning speed of 5 °/min
SEM	Au,0.8nm(15kV), 10kV, 200nA
TEM	60–100kV, DMSO
DSC	5–10 mg, 5/10/15/20 °C/min, N_2
GPC	DMSO
TG	10 °C/min, Air
Xc	ΔH_0=140 J/g

Table S3.

Thermal properties and crystallization kinetics analysis data of LPOBD/PET composites (10 °C/min).

**Samples**	**Tc(°C)**	**Tm(°C)**	**ΔHm(J/g)**	**ΔHc(J/g)**	**Xc(%)**	**$t_{1/2}$(s)**	**n**	**Kc**
Pure PET	182.3	248.7	32.1	31.1	22.2	128	2.35	0.26
0.5%	187.5	250.2	34.5	33.5	24.0	108	2.62	0.31
0.75%	192.4	251.4	35.2	33.7	24.3	102	2.74	0.36
1.0%	199.8	252.5	36.3	34.9	25.2	94	2.76	0.38
1.25%	195.7	251.9	35.8	33.9	24.5	99	2.68	0.35

Table S4.

Thermal properties and crystallization kinetics analysis data of LPOBD−T/PET composites (10 °C/min).

**Samples**	**Tc(°C)**	**Tm(°C)**	**ΔHm(J/g)**	**ΔHc(J/g)**	**Xc(%)**	**$t_{1/2}$（s）**	**n**	**kc**
pure PET	182.3	248.7	32.1	31.1	22.2	128	2.35	0.26
0.5%	189.4	251.3	34.8	33.4	23.9	105	2.77	0.35
0.75%	196.9	253.1	36.2	34.8	25.0	84	2.73	0.39
1.0%	203.4	254.5	37.8	35.3	25.5	75	2.81	0.43
1.25%	198.1	253.4	37.1	34.7	25.1	86	2.84	0.40

Table S5.

The scattering crystallographic plane (hkl), 2θ, dhkl, Lhkl, and xc values of PET materials.

**Composition**	**(*hkl*)**	**2*θ* (°)**	**d_{hkl} (Å)**	**L_{hkl} (nm)**	**x_c (%)**
pure PET	(011)	16.78	5.28	13.5	21.7
	(010)	18.14	4.88	12.3	
	(110)	23.30	3.81	10.6	
	(100)	26.64	3.34	9.8	
1wt% LPOBD/PET	(011)	16.84	5.26	14.1	26.3
	(010)	18.21	4.87	12.7	
	(110)	23.42	3.79	10.9	
	(100)	26.66	3.34	10.2	
1wt% LPOBD−T/PET	(011)	16.92	5.23	14.5	27.5
	(010)	18.17	4.87	13.0	
	(110)	23.46	3.78	10.9	
	(100)	26.72	3.33	10.1	

Table S6.

Thermal properties and crystallization kinetics analysis data of 1wt% LPOBD/PET composites at different cooling rates.

Samples	Tc(°C)	Tm(°C)	ΔHm(J/g)	ΔHc(J/g)	Xc(%)	$t_{1/2}$(s)	n	Kc
5 °C/min	198.5	252.1	31.5	30.0	21.6	125	2.71	0.31
10 °C/min	199.8	252.5	36.3	34.9	25.2	94	2.76	0.38
15 °C/min	201.1	253.7	45.6	43.9	31.7	86	–	–
20 °C/min	200.3	252.9	38.2	35.4	25.5	78	–	–

Table S7.

Thermal properties and crystallization kinetics analysis data of 1wt% LPOBD−T/PET composites.

Samples	Tc(°C)	Tm(°C)	ΔHm(J/g)	ΔHc(J/g)	Xc(%)	$t_{1/2}$（s）	n	kc
5 °C/min	202.7	253.7	32.1	30.2	21.8	106	2.75	0.36
10 °C/min	203.4	254.5	37.8	35.3	25.5	75	2.81	0.43
15 °C/min	204.9	255.8	46.3	44.7	32.2	70	–	–
20 °C/min	203.2	253.9	36.9	35.0	25.3	58	–	–

Table S8.

Thermal properties of hybrid PET materials.

Sample	$T_{10\%}$ (°C)	$T_{50\%}$ (°C)	T_{max} (°C)
pure PET	391.17	426.77	464.82
1% LPOBD/PET	395.41	429.63	471.53
1% LPOBD−T/PET	397.38	433.10	478.46

Table S9.

Abbreviations and their corresponding full names.

Abbreviations	Full names
PET	Poly(ethylene terephthalate)
LMPET	Low molecular weight PET
BATD	1,3–bis(3–aminopropyl)– 1,1,3,3–tetramethyldisiloxane
TEOS	Tetraethyl orthosilicate
OBD	oxalic acid/BATD/oxalic acid
LPOBD	BATD/oxalic acid/LMPET
LPOBD–T	BATD/oxalic acid/LMPET/TEOS

Table S10.

A comparison between the results of this paper and the published results.

Items	1	2	This paper
Material	Nano–SiO_2	Tetraethyl Orthosilicate	1,3–bis(3–aminopropyl)– 1,1,3,3–tetramethyldisiloxane
Optimum dosage	2 wt%	0.75 wt%	1 wt%
The increase in melting temperature	17.7 °C	10.3 °C	5.8 °C
The increase in crystallization temperature	12.3 °C	7.1 °C	21.1 °C
The increase in crystallization	2.3%	8.7%	3.3%
